# Severe Gingival Enlargement with Coexisting Erosive Lichen Planus in Severe Chronic Periodontitis Patient

**DOI:** 10.1155/2015/538538

**Published:** 2015-03-09

**Authors:** Ambika Sharma, Chakshu Aggarwal, Vijay P. Mathur, Divesh Sardana

**Affiliations:** ^1^Department of Periodontology, Centre for Dental Education & Research, AIIMS, New Delhi 110029, India; ^2^Department of Oral Pathology and Microbiology, Centre for Dental Education & Research, AIIMS, New Delhi 110029, India; ^3^Department of Pedodontics and Preventive Dentistry, Centre for Dental Education & Research, AIIMS, New Delhi 110029, India

## Abstract

Plaque induced gingival enlargement is most commonly seen and when encountered simultaneously with erosive lichen planus poses a challenge to the treating dentist. Prognosis of one condition may influence the prognosis of another condition. The presented case highlights the significance of proper diagnosis and the management of simultaneously occurring gingival lesions. A 49-year-old hypertensive female presented with painful enlarged bleeding and suppurating gums with burning sensation on eating food along with long-term usage of antihypertensive drug amlodipine known for its gingival enlargement effect. All these multiple factors led to diagnostic dilemma. Effective management of the gingival enlargement was done by using electrocautery to rehabilitate the functions and esthetics of the patient. Gingival condition was also complicated by the presence of coexisting lichen planus which was predominantly erosive for which topical corticosteroid, antifungal, and antimicrobial agents were prescribed. Eight-month follow-up did not show recurrence of gingival enlargement. Electrocautery is an effective tool for the gingivectomy in severe inflammatory type of gingival enlargement because of rapid postoperative hemostasis. For the management of erosive lichen planus, long-term use of topical corticosteroids is an effective approach. Maintenance of oral hygiene and regular follow-ups are essential for these conditions.

## 1. Introduction

Gingival enlargement, previously known as gingival hyperplasia or gingival hypertrophy, is an increase in the size of gingiva. It is a common feature of gingival diseases. There are several causes of gingival enlargement which can be grouped into five categories: inflammatory, drug induced, in association with systemic diseases or conditions, neoplastic, and false enlargement [1]. Chronic plaque accumulation can also lead to chronic inflammatory gingival enlargement.

Oral lichen planus (OLP), the mucosal counterpart of cutaneous lichen planus, presents frequently in the fourth decade of life and affects women more than men in a ratio of 1.4 : 1 [2]. OLP can be seen clinically as reticular, papular, plaque-like, erosive, atrophic, or bullous types. Atrophic lesions account for 5% to 44% of OLP manifestations, while the erosive and/or ulcerative ones vary between 9% and 46% of cases [3]. Most of the subjects with erosive forms of lichen planus (LP) present with symptoms of pain and burning sensation in the affected area.

Gingival manifestation of multiple diseases/conditions may lead to difficulty in diagnosis as well as in management as seen in this reported case where multiple conditions like chronic periodontitis, gingival enlargement, erosive lichen planus, history of long-term use of drug Amlodipine known for causing drug induced gingival enlargement [4], and history of menopause were present.

An understanding of the cause and underlying pathologic changes is essential for the treatment of gingival enlargement. In the present case, patient had severe chronic inflammatory gingival enlargement with coexisting lichen planus which was predominantly erosive manifested as desquamative gingivitis with underlying severe chronic periodontitis. This case was treated effectively in the following phases: (1) through phase 1 therapy including supra- and subgingival scaling along with prescription of 0.2% chlorhexidine mouth rinse for two weeks and patient motivation and education, (2) substitution of the drug Amlodipine with drug Telmisartan, (3) surgical excision of the residual gingival overgrowth with the help of electrocautery under local anesthesia, and (4) maintenance and supportive therapy.

Electrocautery was used at 50 Hz frequencies at electrosection and electrocoagulation modes. For the management of erosive lichen planus, topical corticosteroids including Kenacort oral paste (0.1% triamcinolone acetonide) and mouth rinse comprising 0.5 mg betamethasone were prescribed. Antifungal agent in the form of candid mouth-paint (1% w/v clotrimazole) was also prescribed to prevent opportunistic candida infection.

## 2. Case History

A 49-year-old female patient presented with chief complaint of swollen gums in upper front region. She first noticed a small painless growth from gingiva about a year back. The size of the growth did not increase much during the first six months; however, there was a rapid increase in the later six months. She also had multiple mobile teeth, halitosis, sticky discharge, and unprovoked bleeding from the gums during the last few months. The patient also had history of burning sensation while eating food and difficulty in mastication, speech, and brushing teeth. She did not have previous diagnosis and treatment for this burning sensation. Medical history of hypertension was present for which she was prescribed antihypertensive drug, Tab. Amlodipine (long-acting dihydropyridine) 5 mg/day, to be administered systemically once a day orally for the last 12 years.

On the day of reporting to the hospital, she was conscious, cooperative, and with no visible gross problems. However, the lips were incompetent and enlarged gingiva was protruding between the lips. Apart from this, no extraoral swelling or cutaneous lesions were observed.

**Figure 1:**
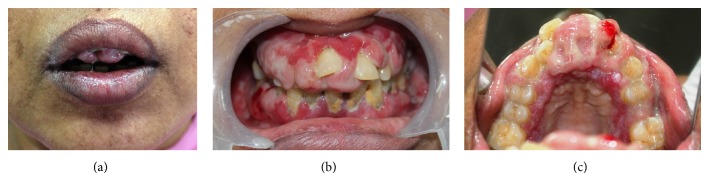
(a) shows incompetent lips due to gingival overgrowth. (b) and (c) show intraoral gingival enlargement with erythematous lesion, spontaneous bleeding sites, calculus, and purulent discharge.

**Figure 2:**
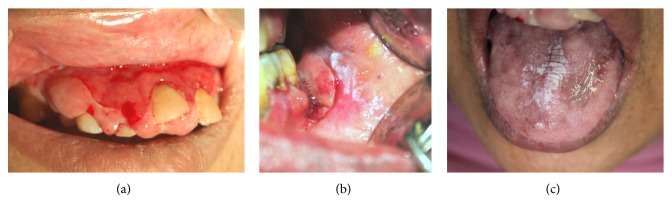
(a) shows generalized erythema and fragility with very fine white striae at the tips of interdental papillae. (b) shows dense white striae (plaque type LP) bordering erythematous lesions on buccal mucosa. (c) shows white plaque type patch with loss of papilla and melanin pigmentation on the dorsum of tongue.

Intraoral examination revealed diffuse gingival enlargement which was massive in size in maxillary anterior region extending from right second premolar to left second premolar. Enlargement of the gingiva was in the horizontal as well as in the vertical direction protruding below the incisal edges of the anterior teeth on palatal aspect. Diffuse moderate gingival enlargement was also present on other regions of the upper arch and on the lower arch (Figure 1(a) shows incompetent lips due to gingival swelling; Figures 1(b) and 1(c) show the extent of intraoral gingival swelling). This was soft and edematous on palpation with high tendency to bleed even on gentle touch. Generalized diffuse desquamative gingivitis along with creamy striae on the periphery of desquamative gingival lesions was noted. Bilateral white lesions with striae at borders were noted on buccal mucosa and dorsum of tongue which were denuded of papilla (Figure 2(a) shows desquamative gingivitis; Figures 2(b) and 2(c) show plaque type lichen planus on buccal mucosa and tongue). Periodontal probing revealed presence of true periodontal pockets with attachment loss of more than 3 mm in most of the teeth in maxillary arch. Attachment loss of more than 5 mm along with pathological migration was noted in maxillary central and lateral incisors. Grade 2 mobility was noticed in both maxillary central incisors and grade 1 mobility in the mandibular incisors. Spontaneous bleeding and purulent discharge were present from gingival sulcus of most of the teeth. Based on these clinical findings, provisional diagnosis of severe chronic periodontitis was concluded with presentation of severe inflammatory gingival enlargement and lichen planus (LP) which was predominantly erosive in nature. These were coexisting conditions at the same time. Role of drug induced gingival enlargement was also suspected in the present case, as the patient was taking Tab. Amlodipine to control hypertension for the last 12 years, which is known to cause fibrotic gingival enlargement.

**Figure 3:**
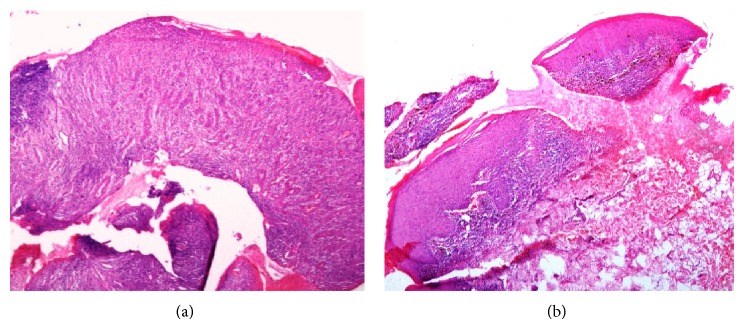
(a) shows localized band-like inflammatory cell infiltrate in the connective tissue and ulcerated surface epithelium with thin endothelium-lined capillary structures and inflammatory cells. (b) shows thickening of surface epithelium with basal cells vacuolization, dense lymphocytic infiltrate at epithelium connective tissue junction with serrated rete ridge pattern.

Orthopantomograph revealed generalized horizontal pattern of bone loss. Routine blood investigations, including complete blood count, blood sugar test, bleeding time, and clotting time, were performed, which were found to be in normal range. Two incisional biopsies, one from palatal gingiva and another from left buccal mucosa, were obtained under local anaesthesia for the histopathological examination. Histopathological evaluation of gingival region biopsy revealed localized band-like dense inflammatory infiltrate with numerous capillary structures lined by plump endothelial cells, moderately scattered inflammatory cells in connective tissue with loss of surface epithelium compatible with erosive type of lichen planus, and pyogenic granuloma (Figure 3(a)). Histopathological evaluation of buccal mucosal lesion showed thickening of parakeratinized stratified squamous epithelium, degenerating basal cells with vacuolization, and dense lymphocytic infiltrate at epithelium connective tissue junction. Saw tooth rete ridge pattern and subepithelial histological clefts were also seen (Figure 3(b)). Confirmatory diagnosis of LP was rendered (which was plaque type in clinical presentation).

**Figure 4:**
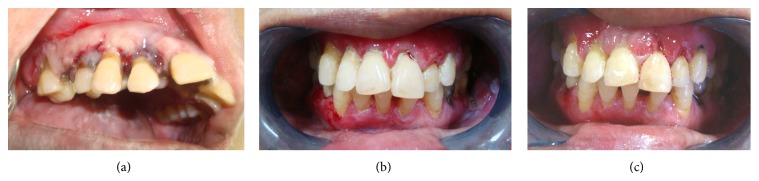
(a) shows follow-up after 1 week with initial healing. (b) shows healing after one month. (c) shows gingival status on 8-month follow-up.

Before starting dental treatment, patient was referred to her treating physician for the substitution of drug Amlodipine, due to its known etiological role with another antihypertensive drug. Tab. Amlodipine was then substituted with Tab. Telmisartan 40 mg. Patient was motivated and educated for oral hygiene maintenance by instructing proper brushing technique. Treatment was started with nonsurgical phase 1 therapy which included gross scaling and root planning with ultrasonic scalers. 10 mL of 0.2% chlorhexidine gluconate mouthwash (which provides 20 mg of effective dose against plaque formation) was prescribed twice daily for two weeks. Only mild reduction was noted in signs and symptoms of gingival inflammation after 14 days of oral prophylaxis. Because of persisting inflammatory nature of the lesion, its massive size, and high bleeding tendency, excision of the enlarged gingival tissue was planned with the help of electrocautery. Debulking incisions were given for the gingivectomy at 50 Hz frequencies with needle electrode. Patient was prescribed nonsteroidal anti-inflammatory analgesic Tab. Flexon (ibuprofen 400 mg and paracetamol 500 mg) twice a day for three days, and to prevent postoperative infection, broad spectrum antibiotic effective against most of the pathogenic oral Gram-positive and Gram-negative bacteria, Cap. amoxicillin 500 mg 6 hourly for one week, was also prescribed. At 7-day follow-up visit (Figure 4(a)), no postoperative complication was encountered and healing was found to be uneventful. After 15 days, complete oral prophylaxis was done and oral hygiene instructions were reinforced. Consultation from dermatology and oral medicine department was taken by us for the first time for their expert opinion regarding treatment of erosive lichen planus. Patient was then prescribed topical corticosteroids, Kenacort oral paste (0.1% triamcinolone acetonide), and mouth rinse comprising 0.5 mg betamethasone along with antifungal agent candid mouth-paint (1% w/v clotrimazole) twice a day for one month. Patient was recalled after every week for the first month and then every month (Figure 4(b) after one month) for follow-ups and oral prophylaxis. No recurrence of gingival enlargement was seen after 8-month follow-up (Figure 4(c)) but with mild erythematous lesions. Patient was able to perform her routine functions normally. Patient is still under the treatment for erosive lichen planus with regular follow-ups.

## 3. Discussion

The gingival enlargement in the reported case was of the inflammatory type. The exact role of Amlodipine was not predictable in the present case which is known to cause fibrotic gingival enlargement. Clinical manifestation of drug induced gingival enlargement frequently appears within 1 to 3 months after initiation of treatment with the associated medication [5]. But in the reported case, patient was taking Amlodipine for the last 12 years and noticed the enlargement since the last year only. Moreover, clinical and histological picture mimic inflammatory lesion instead of fibrotic drug induced gingival enlargement. In this case, chronic plaque accumulation which led to chronic periodontitis appeared to be the etiological agent for the inflammatory type of gingival enlargement. Hormonal changes due to menopause appeared to contribute further to the inflammation of the gingival tissue [6]. Concomitant presence of oral lichen planus which is also a chronic inflammatory disease has increased the inflammatory component. Impeded maintenance of oral hygiene practice because of bleeding tendency and increased size of gingiva led to the formation of vicious cycle for increased plaque accumulation and thus further increase in gingival inflammation. Commonly involved sites for OLP are the buccal mucosa, tongue, and the gingiva although other sites may be affected. The erosive form primarily manifests in the buccal mucosa and lingual dorsum, and it, along with the atrophic form, appears with most of the symptoms [7].

The diagnosis of OLP is based on the clinical appearance of the lesions and is subsequently confirmed by histopathological study [8]. In this case, diagnosis of LP was made by us for the first time by its clinical and histological evaluation. The presence of erosive lichen planus on the gingival mucosa is characterized by the presence of diffuse erythematous areas that may or may not be interspersed with desquamative and ulcerated foci. The whitish lesions may occur following the shape of the gingival outline. Characteristic hyperkeratotic radiating striae found at the periphery of the erosive regions also aid in diagnosis as observed in the present case. The clinical appearance known as desquamative gingivitis is not pathognomonic of oral erosive lichen planus and may represent the gingival manifestation of many other diseases such as pemphigus vulgaris, cicatricial pemphigoid, epidermolysis bullosa acquisita, lupus erythematosus, and linear IgA disease.

Several antigen-specific and nonspecific inflammatory mechanisms have been put forward to explain the pathogenesis of OLP. It is considered as T-cell-mediated autoimmune disease in which the autocytotoxic CD8+ T cells trigger apoptosis of the basal cells of the oral epithelium [9]. The migrated CD8+ cells are activated directly by antigen binding to major histocompatibility complex (MHC-1) on keratinocyte or through activated CD4+ lymphocytes. The matrix metalloproteinase (MMP) is principally involved in tissue matrix protein degradation. MMP-9, which cleaves collagen 4 along with its activators, is upregulated in OLP lesional T cells, resulting in increased basement membrane disruption [10].

There is controversy regarding malignant potential of OLP. Some studies indicate an increased risk of squamous cell carcinoma in patients with OLP lesions [11, 12]. A review of previously published studies concluded that the risk of developing squamous cell carcinoma in patients with OLP is approximately ten times higher than that in the unaffected general population [13].

It has been commonly associated with the erosive and atrophic forms [3, 14]. Most cases of reported malignant transformation are rather poorly documented. Some of these cases may not have been true lichen planus, but rather they may have actually been dysplastic leukoplakia with secondary lichenoid inflammatory infiltrate which mimicked lichen planus [5].

In contrast, other studies rule out the connection between OLP and oral cancer [15, 16]. Despite the controversy, clinician should perform biopsy and close follow-up in these patients.

Hormonal dysfunction, candidosis, lichenoid lesions, and the vulvovaginal-gingival syndrome must also be included in the differential diagnosis of oral erosive lichen planus [17–19]. Difficulties in the establishment of diagnosis of gingival lichen planus may arise if gingivitis and periodontitis are superimposed on the lesions [8]. As in the present case, periodontitis with severe gingival enlargement and superimposed erosive lichen planus were present simultaneously and led to diagnostic dilemma.

Treatment is aimed primarily at reducing the length and severity of symptomatic outbreaks. The most widely accepted treatment for OLP is topical corticosteroid because they are directly applied over the affected area and have less chance of systemic complications. These are the single most useful group of drugs having the ability to modulate inflammation and immune response. Alternative treatments like retinoid, cyclosporine, tacrolimus, surgery, and carbon dioxide (CO_2_) laser may also be used. Recently, low level laser therapy (LLLT) has been used for treating erosive OLP with minimal side effects [20–22]. Treatment choice may vary from patient to patient depending on their symptomatic history, severity of lesions, and systemic condition of the patient. Excellent oral hygiene is also required to reduce the severity of the symptoms, but it can be difficult for patients to achieve high levels of hygiene during periods of active disease. Currently, there is no definitive cure for OLP. Unlike cutaneous lichen planus, which in most cases progresses by short-term outbreaks that almost always respond well to treatment or even regress after a few months, OLP is characterized by its chronicity, persistence with periods of exacerbation and quiescence, and resistance to therapy.

In the present case, topical corticosteroids, antifungal agents, and antimicrobial mouth rinse were prescribed. Kenacort oral paste (0.1% triamcinolone acetonide) and mouth rinse prepared by dissolving Tab. Betnesol 0.5 mg (betamethasone) in 10 mL of water were prescribed after consultation with oral diagnostician. Since prolonged use of corticosteroids can cause opportunistic infection like oral candidiasis or thrush, antifungal agent as Candid gel (clotrimazole 1% w/v) was also prescribed to the patient for twice daily application. Patient was motivated and educated for maintaining good oral hygiene.

For patients with gingival overgrowth, the modification of tissue topography by surgical recontouring or gingivectomy may be undertaken to create a maintainable oral environment [17]. In the present case, treatment was largely limited to the maintenance of an improved level of oral hygiene by doing oral prophylaxis at every recall visit and surgical removal of the overgrowth tissues. Since the lesion was predominantly inflammatory in nature, electrocautery was used for the gingivectomy procedure. Electrocautery permits an adequate contouring of the tissue and controls hemorrhage [23, 24]. Patient was relieved symptomatically for OLP but not cured completely but was able to perform the routine functions and is aesthetically and psychologically satisfied.

## 4. Conclusion

Proper diagnosis and management are essential when multiple diseases or conditions manifest in the oral cavity as seen in the present case. Electrocautery is effective means of gingivectomy in severely inflamed, massive sized gingival enlargement with marked tendency for bleeding.

Topical steroids are safer and effective means for the palliative treatment of erosive lichen planus. Routine follow-up with maintenance of good oral hygiene is also an important part of management of gingival lesions.
